# Supplementary material for: Endophytic Microorganisms in Tomato Roots, Changes in the Structure and Function of the Community at Different Growing Stages
Source: Microorganisms. 2024 Jun 20;12(6):1251. doi: 10.3390/microorganisms12061251 (PMC11206058; doi:10.3390/microorganisms12061251)
Supplement: Supplementary file 1 [file microorganisms-12-01251-s001.zip › microorganisms-2996270-supplementary.pdf]

Supplementary Materials

Table S1. Sequencing information of bacteria in root of tomatoes under different growth stages.

Sample\Info	Seq_num	Base_num	Mean_length	Min_length	Max_length
GH1	24770	9310531	375.87933	202	443
GH2	24193	9099384	376.116397	200	488
GH3	23092	8681861	375.968344	204	515
GJ1	22316	8392293	376.066186	203	467
GJ2	20282	7619816	375.693521	207	478
GJ3	24620	9277244	376.817384	202	489
GY1	21760	8178415	375.846278	201	458
GY2	23931	8997021	375.956751	206	528
GY3	24095	9054416	375.779871	200	424

Table.S2 Sequencing information of fungi in root of tomatoes under different growth stages

Sample\Info	Seq_num	Base_num	Mean_length	Min_length	Max_length
GH1	73910	15475450	209.382357	143	504
GH2	70784	14970472	211.49514	143	495
GH3	73980	15506775	209.607664	145	530
GJ1	73167	15293170	209.017317	166	496
GJ2	73058	16099065	220.360056	143	526
GJ3	74588	16001512	214.531989	140	523
GY1	52322	12417391	237.326383	159	530
GY2	57439	16048810	279.406153	193	495
GY3	71738	15863673	221.133472	147	484

Table. S3 Amplified sequence information of tomato root bacteria and fungi under different growth stages

Type	Amplified Region	Insert size(bp)	Raw Reads	Sequences	Bases (bp)	Total Base(bp)	Average Length
Bacteria	799F_1193R	394	209059*2	209059	78610981	125853518	376
Fungi	ITS1F_ITS2R	300	1234928*2	1234928	287046596	743426656	232
